# Oxidative stress tolerance contributes to heterologous protein production in *Pichia pastoris*

**DOI:** 10.1186/s13068-021-02013-w

**Published:** 2021-07-20

**Authors:** Nai-Xin Lin, Rui-Zhen He, Yan Xu, Xiao-Wei Yu

**Affiliations:** Key Laboratory of Industrial Biotechnology, Ministry of Education, School of Biotechnology, Jiangnan University, Wuxi, 214122 People’s Republic of China

**Keywords:** Yeast, *Pichia pastoris*, Heterologous protein expression, Lipase, Oxidative stress tolerance

## Abstract

**Background:**

*Pichia pastoris* (syn. *Komagataella phaffii*) is an important yeast system for heterologous protein expression. A robust *P. pastoris* mutant with oxidative and thermal stress cross-tolerance was acquired in our previous study. The robust mutant can express a 2.5-fold higher level of lipase than its wild type (WT) under methanol induction conditions.

**Results:**

In this study, we found that the robust mutant not only can express a high level of lipase, but also can express a high level of other heterogeneous proteins (e.g., green fluorescence protein) under methanol induction conditions. Additionally, the intracellular reactive oxygen species (ROS) levels in the robust mutant were lower than that in the WT under methanol induction conditions. To figure out the difference of cellular response to methanol between the WT and the robust mutant, RNA-seq was detected and compared. The results of RNA-seq showed that the expression levels of genes related to antioxidant, MAPK pathway, ergosterol synthesis pathway, transcription factors, and the peroxisome pathway were upregulated in the robust mutant compared to the WT. The upregulation of these key pathways can improve the oxidative stress tolerance of strains and efficiently eliminate cellular ROS. Hence, we inferred that the high heterologous protein expression efficiency in the robust mutant may be due to its enhanced oxidative stress tolerance. Promisingly, we have indeed increased the expression level of lipase up to 1.6-fold by overexpressing antioxidant genes in *P. pastoris*.

**Conclusions:**

This study demonstrated the impact of methanol on the expression levels of genes in *P. pastoris* and emphasized the contribution of oxidative stress tolerance on heterologous protein expression in *P. pastoris*. Our results shed light on the understanding of protein expression mechanism in *P. pastoris* and provided an idea for the rational construction of robust yeast with high expression ability.

## Background

With its proven ability to express over 400 proteins, from human endostatin to spider dragline silk protein, *Pichia pastoris* has become a consistent choice for heterologous protein production [1–3]. Additionally, *P. pastoris* as a eukaryotic organism is capable of secreting high titres of correctly folded, post-translationally processed and active recombinant proteins into the culture media [4, 5]. *P. pastoris* has the ability to produce proteins of therapeutic and commercial interest in concentrations ranging from milligrams to grams per litre [6]. Fermentations can be readily scaled up to meet greater demands, and factors influencing protein productivity and activity, can be controlled. Furthermore, as *P. pastoris* does not secrete high levels of native proteins, the purification of secreted makings is much easier than in other systems [7].

Many methods have been used to improve the expression of heterologous proteins in *P. pastoris*, mainly by codon optimization, increasing gene dosage, promoting protein folding and secretion, etc. Codon usage differs among organisms, thereby substituting rare codons basing on the preferred codon usage of *P. pastoris* is vital to realize high expression levels of heterologous proteins. The efficient production of a novel pH-stable xylanase from the fungus *Corynascus thermophilus* was achieved by codon optimization [8]. Simply increasing the copy number of target genes can improve the production of target heterologous proteins [9, 10]. However, the overexpression of heterologous protein might cause the accumulation of unfolded and misfolded protein in the endoplasmic reticulum (ER), activating the unfolded protein response (UPR) [11]. After that, the ER-associated degradation pathway can be activated and then reduce the production of heterologous proteins. Based on the above rationales, to prevent the degradation of overexpressed heterologous protein by the proteasome, the disruption of Pep4 protease increases phytase secretion in *P. pastoris* [12]. Lipase is widely used in the production of biodiesels [13], detergents [14], chiral compounds [15], etc. Protein disulfide isomerase (PDI), a chaperone in ER, can catalyse the disulfide bond formation and help in correcting the folding of a protein. Previously, we successfully improved the expression level of lipase in *P. pastoris* by co-expressing PDI [16].

During fermentation process, cells may encounter multiple environmental stresses from the fermentation medium, product, temperature, etc. These environmental stresses not only reduce the production of products, but even limit the growth of yeast cells. As we know, H_2_O_2_ generated by the metabolism of methanol may cause oxidative stress and impact the production efficiency of *P. pastoris*. However, research had barely reported the effect caused by methanol on the production of heterologous protein and the change of cellular response in *P. pastoris*. Additionally, few researchers have tried to strengthen the heterologous protein expression ability in *P. pastoris* from the perspective of improving their oxidative stress tolerance. Indeed, many other researchers have successfully increased the production efficiency of yeast strains by relieving repression and improving their stress tolerance. These examples provide a good reference for the modification in *P. pastoris*. For example, researchers have greatly improved the ethanol production of a *Kluyveromyces marxianus* mutant by adaptive evolution in 6% (v/v) ethanol [17]. The glucose and xylose fermentation in *Saccharomyces cerevisiae* under acetic acid stress conditions was improved by overexpressing *RCK1* coding for a protein kinase involved in oxidative stress [18].

Previously, a *P. pastoris* mutant with thermal and oxidative stress cross-tolerance was found to have a higher lipase expression ability than its WT under methanol induction conditions. In this study, we found that this phenomenon was also present in the expression of other protein. Given the high oxidative stress tolerance in the robust mutant, we examined the changes of the intracellular reactive oxygen species (ROS) levels in the WT and the robust mutant under methanol-induced conditions. Additionally, the transcriptomes of the WT and the robust mutant during the methanol-induced fermentation process and growth condition were also explored and compared. As a result, we found that the mechanism of high heterologous protein expression ability in the robust mutant was mainly owed to its enhanced oxidative stress tolerance. To validate this inference, we rationally designed antioxidant gene-overexpressed strains, and successfully improved the heterologous protein expression ability in these strains compared to their blank control. This study emphasized the importance of oxidative stress tolerance in protein expression in *P. pastoris* and provided ideas for rationally increasing the production efficiency of heterologous protein in the future.

## Results

### Improved heterologous protein expression ability in the robust mutant under methanol induction

**Fig. 1:**
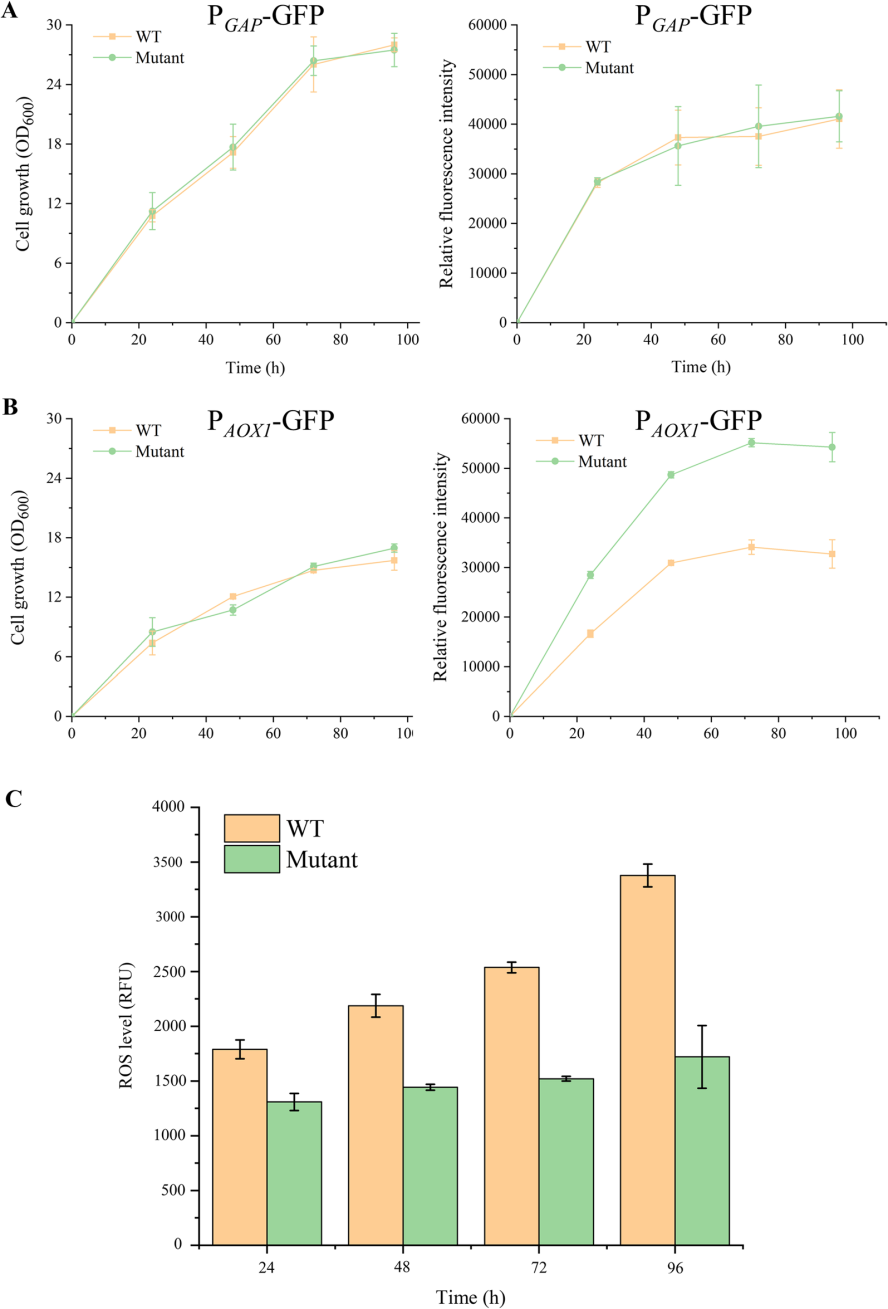
The characteristics of the WT and the robust mutant. **A** The cell growth and fluorescence intensity of green fluorescence protein (GFP) in the WT and the robust mutant under glucose condition; **B** the cell growth and fluorescence intensity of GFP in the WT and the robust mutant under methanol condition; **C** ROS levels of the WT and the robust mutant during fermentation process under the induction of methanol

Previously, we isolated a robust *P. pastoris* mutant, which exhibited thermal and oxidative co-stress tolerance. In addition, the robust mutant expressed an up to 2.5-fold higher level of heterologous protein than the WT [19]. To determine whether this phenomenon could also occur in the expression of other proteins, a green fluorescence protein (GFP) was expressed in the robust mutant and its WT. Two promoters, P_*GAP*_ and P_*AOX1*_, were used to initiate the expression of GFP. The alcohol oxidase 1 (AOX1) promoter (P_*AOX1*_) of *P. pastoris* is one of the most widely used promoters for expressing a large number of proteins. The action of P_*AOX1*_ is intensely repressed by multiple carbon sources such as glucose and glycerol, but is strongly induced by methanol [20]. P_*GAP*_ is a common strong constitutive promoter from the glyceraldehyde 3-phosphate dehydrogenase gene (GAP) [21]. Under the control of P_*GAP*_, the expression levels of GFP were similar in the WT and the robust mutant (Fig. 1A). However, under the control of P_*AOX1*_ with methanol as an inducer, the expression level of GFP in the robust mutant was 1.3-fold higher than that of in the WT (Fig. 1B). In addition, the cell growth curve showed that the growth of the WT and the robust mutant under methanol condition was inhibited compared with that under glucose condition. The results of GFP expression in the WT and the robust mutant indicated that the robust mutant expressed a higher level of heterologous proteins under methanol induction. We inferred that the robust mutant might be more capable to overcome the oxidative stress caused by methanol than its WT, leading to its enhanced protein expression ability. We then detected the intracellular ROS levels of the robust mutant and the WT and tried to analyse the expression mechanism of the robust mutant and the WT by RNA-seq.

### Enhanced anti-oxidative stress ability helps to reduce the accumulation of ROS in *P. pastoris*

Under the control of P_*AOX1*_, methanol is used as an inducer and a sole carbon resource for fermentation with *P. pastoris*. The metabolism of methanol generates hydrogen peroxide (H_2_O_2_), which is a kind of ROS [22]. During the fermentation process under the induction of methanol, the intracellular ROS levels of WT and the robust mutant were measured and compared (Fig. 1C). The accumulation of ROS in cells gradually increased over time. However, the accumulations of ROS in the WT were higher up to twofold than that in the robust mutant. The result indicated that a stronger ROS buffering system may be in the robust mutant to efficiently reduce the ROS level caused by methanol. Hence, RNA-seq of the robust mutant and the WT was carried out to further explore the underlying mechanism.

### Augmented ROS buffering capacity renders higher expression ability based on transcriptome analysis

**Fig. 2:**
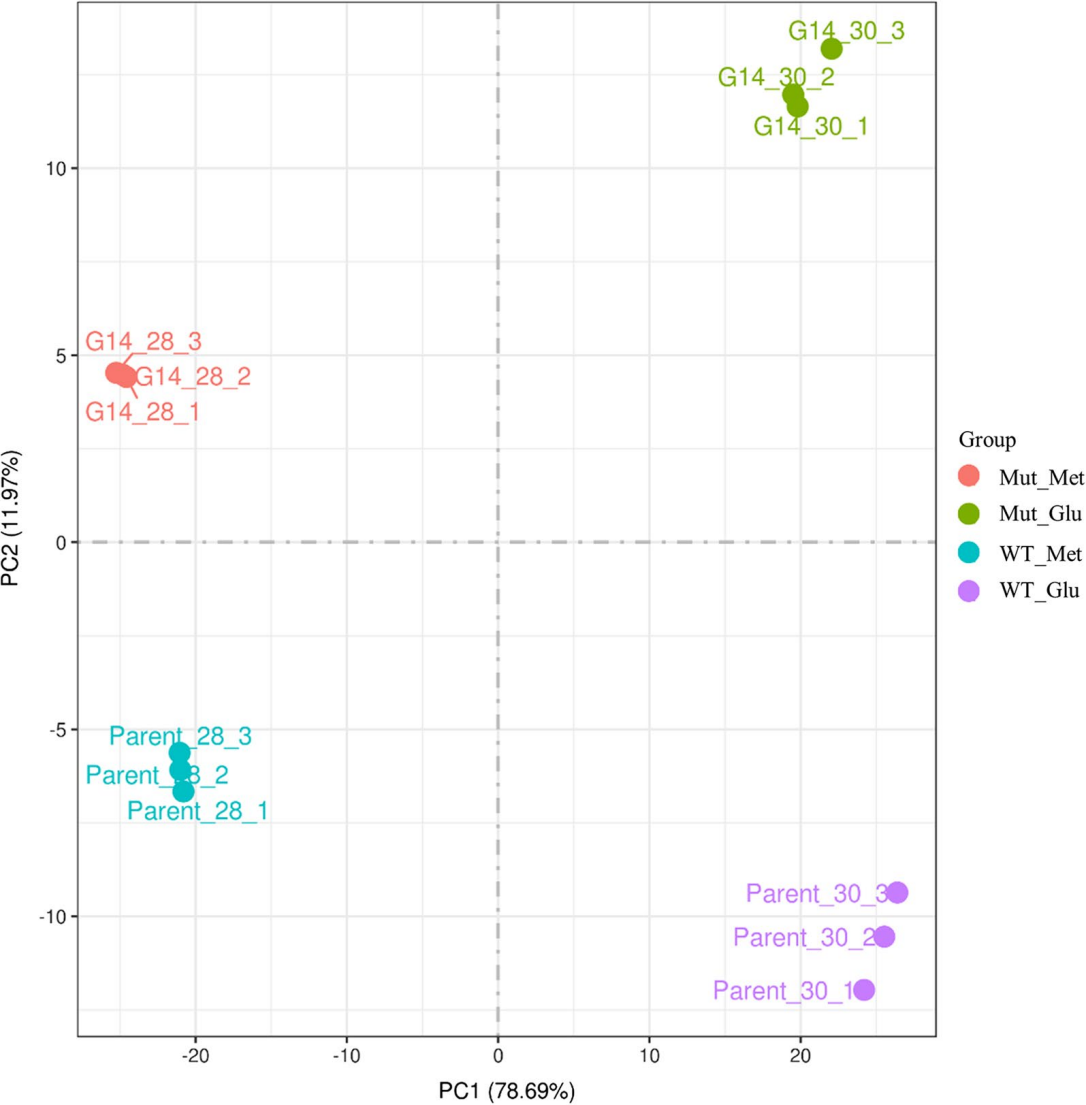
The correlation of inter-group samples by PCA analysis. Three biological replicates were used in each group

The heterologous protein expression ability of *P. pastoris* can mainly be mediated by cellular stress, transcription, protein folding, secretion pathway, and carbon metabolism pathway. To explore the underlying mechanism, the transcriptomes of the WT and the robust mutant were analysed and compared under methanol-induced conditions. Yeast cells were collected at 60 h for RNA-seq, as enzyme activity of these *P. pastoris* strains sharply increased at 60 h. The data of RNA-seq was been uploaded in NCBI’s Gene Expression Omnibus (GEO) public archive database and the Accession number is GSE167141. As shown in Fig. 2, the principal-component analysis (PCA) of transcriptome data showed that the triplicates of each sample were similar, indicating that the data could be employed for the following analysis.

**Fig. 3:**
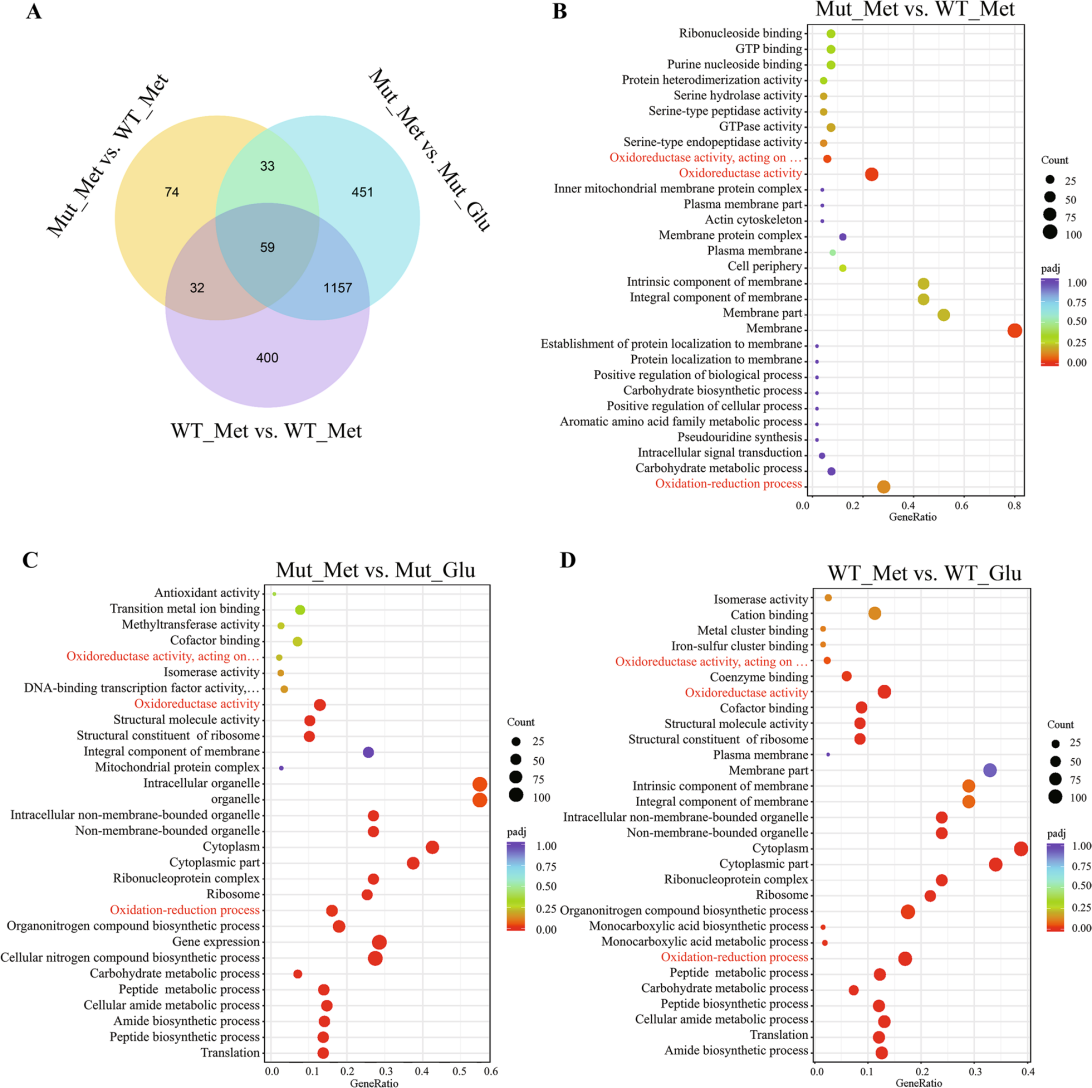
Transcriptomic analysis. **A** The Venn diagram of differentially expressed genes in three comparison groups. **B**–**D** Bubble diagrams of GO enrichment result, each ten GO terms from bottom to top represents biological process, cellular component, and molecular function, respectively. **B** GO enriched analysis of comparison group Mut_Met vs. WT_Met; **C** GO enriched analysis of comparison group Mut_Met vs. Mut_Glu; **D** GO enriched analysis of comparison group WT_Met vs. WT_Glu. The GO terms marked in red include oxidation–reduction process (GO:0055114), oxidoreductase activity (GO:0016491), oxidoreductase activity, acting on CH–OH group of donors (GO:0016614), and oxidoreductase activity, acting on paired donors, with incorporation or reduction of molecular oxygen (GO:0016705)

The analysis of differentially expressed genes was focused on the WT and the robust mutant under methanol induction conditions and glucose culture conditions (previously uploaded in NCBI’s GEO, GSE157242). Three comparison groups were set to explore the cellular response to methanol in *P. pastoris* strains (Fig. 3). The comparison group Mut_Met versus WT_Met represented the robust mutant under methanol condition versus the WT under methanol condition. The comparison group Mut_Met versus Mut_Glu represented the robust mutant under methanol condition versus that in glucose condition. The comparison group WT_Met versus WT_Glu represented the WT under methanol condition versus that in glucose condition. With the absolute value of log_2_-fold change > 1 and *p*-value < 0.05 as classification criteria, the number of differentially expressed genes of each comparison group are listed Venn diagram (Fig. 3A). In the comparison group Mut_Met versus WT_Met, 198 genes were differentially expressed. Methanol caused stress on the WT and the robust mutant, so that a large number of differentially expressed genes were found in the other two comparison groups. A Gene Ontology (GO) enrichment analysis of differentially expressed genes in each comparison group was performed to identify enriched GO terms, including biological process (BP), cellular component (CC), and molecular function (MF). Interestingly, the biological process of oxidation–reduction and the molecular function of oxidoreductase activity were enriched in all these three comparison groups (Fig. 3B–D). The result demonstrated that the oxidative stress caused by methanol may lead to active intracellular redox changes. Moreover, the robust mutant has an enhanced oxidative stress tolerance. The enrichment of GO terms related to redox in comparison group Mut_Met versus WT_Met may explain the reduced ROS levels in the robust mutant.

**Fig. 4:**
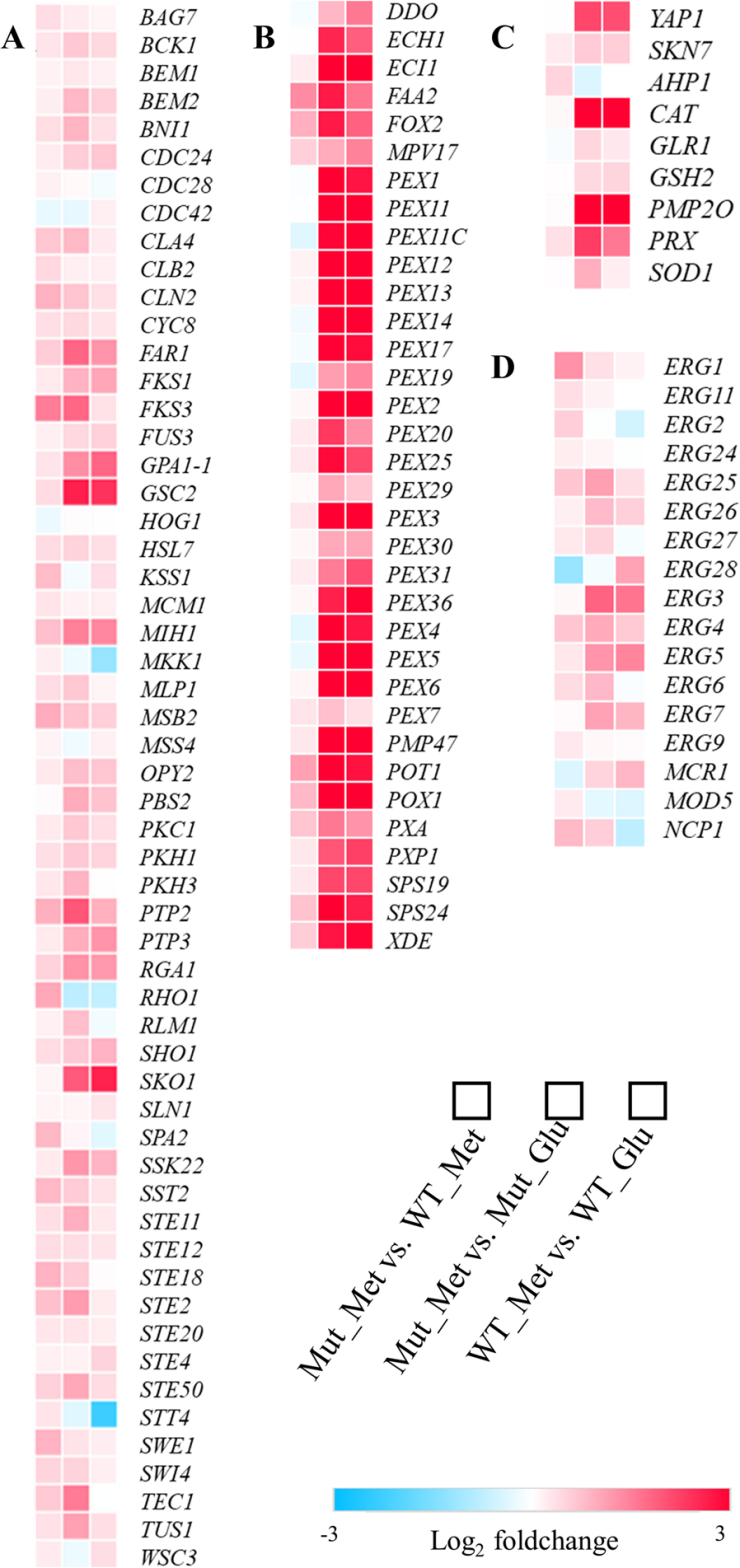
Heat map profiles of key genes. **A** Genes related to MAPK pathway; **B** genes related to peroxisome; **C** genes related to antioxidants and transcription factors responded to oxidative stress; **D** the ergosterol synthesis pathway

The factors related to redox mainly include MAPK pathway, peroxisome, stress response transcription factors, antioxidant defence system, and ergosterol synthesis pathway, so we then investigated the expression levels of related genes (Fig. 4). The MAPK pathway is responsible for resisting environmental stresses [23]. Interestingly, the MAPK pathway (Fig. 4A) was upregulated in all three comparison groups, further indicating methanol is a kind of stress to *P. pastoris*. Furthermore, the upregulation phenomenon was obvious in the comparison group Mut_Met versus WT_Met, indicating that the environmental stress response in the robust mutant was stronger than that in the WT.

The peroxisome pathway (Fig. 4B) was tremendously upregulated in the comparison group Mut_Met versus Mut_Glu and WT_Met versus WT_Glu as the first step of methanol metabolism is in peroxisomes [24]. Peroxisomes possess enzymes that degrade ROS, especially catalase [25]. Predictably, the expression levels of these peroxisomal protein encoding genes were also considerably upregulated in the robust mutant versus the WT under identical methanol conditions, explaining the higher protein expression level in the robust mutant.

Because of the stressful condition caused by methanol, the expression levels of two oxidative stress transcription factors, *YAP1* and *SKN7*, were upregulated in these three comparison groups. The expression levels of several genes related to ROS scavengers (Fig. 4C) were tremendously upregulated in all comparison groups, especially *CAT* (encoding catalase) and *PMP20* (encoding a glutathione peroxidase). Catalase is responsible for removing H_2_O_2_ [26], while *PMP20* is contributed to the removal of other oxidized molecules [27]. The results indicated that more ROS need to be eliminated in the WT and the robust mutant under methanol condition than that in glucose condition. Interestingly, the ergosterol synthesis pathway (Fig. 4D) was showing upregulation in these comparison groups. Other researchers [28] proved that the addition of ergosterol can repress the production of ROS by the NADPH oxidase (NOX). Interestingly, from the result of RNA-seq, the expression level of NOX in the robust mutant is 15% lower than that in the WT. Consistently, most genes related to the ergosterol synthesis pathway in comparison group Mut_Met versus WT_Met were upregulated, further explaining the reduced ROS level in the robust mutant.

### The upregulation of *Mxr1* and *Prm1* improves the efficiency of the AOX1 promoter

**Fig. 5:**
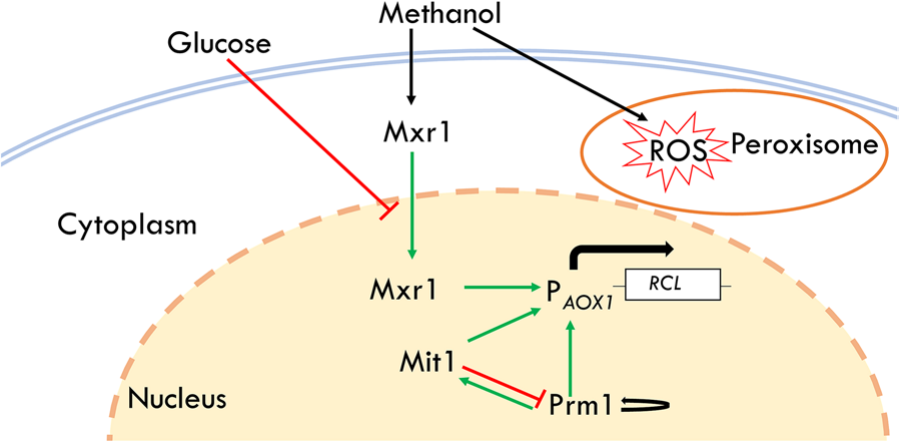
The predicted regulatory model of P_*AOX1*_ activation. Green arrows indicate activation; red blunt-end arrows indicate repression

P_*AOX1*_ is commonly used to initiate the transcription of heterologous proteins in *P. pastoris*, and its transcription efficiency is mediated by transcription factors (TFs) (Fig. 5). Binding sites for TFs, methanol-induced transcription factor 1 (encoded by *Mit1*) [29, 30], methanol expression regulator (encoded by *Mxr1*) [31] and a positive transcription regulator for methanol-utilization genes (encoded by *Prm1*) [32], had been identified on the sequence of P_*AOX1*_. These TFs positively regulate the function of P_*AOX1*_. The transcription levels of *Mit1*, *Mxr1*, *Prm1*, and heterologous lipase gene (*RCL*) were measured and compared between the WT and the robust mutant. The expression levels of *RCL*, *Mxr1*, and *Prm1* were markedly improved in comparison group Mut_Met versus WT_Met according to the results of RNA-Seq and RT-qPCR (Table 1). The expression level of *RCL* was up to 4.2-fold in the robust mutant than that in WT, explaining the higher lipase activity in the robust mutant. The expression level of transcription factors *Mxr1* and *Prm1* in the robust mutant was up to 1.6-fold and 1.8-fold higher than that in the WT, respectively. Therefore, the higher expression level of heterologous protein in the robust mutant may be partly due to the improved efficiency of P_*AOX1*_.

**Table 1:** The expression levels of genes related to the activation of P_*AOX1*_ by RNA-Seq and RT-qPCR

Gene name	Foldchange (RNA-seq)	Adjusted *p*-value	Foldchange (RT-qPCR)	*p*-value	
*RCL*	3.7	***	4.2	*	
*Mxr1*	1.2	***	1.6	**	
*Prm1*	1.2	***	1.8	**	
*Mit1*	0.98	ns	1.2	ns	

Results were means of three biological replicate experiments

*ns* not significant

*p < 0.05; **p < 0.01; ***p < 0.001

### The downregulation of the methanol dissimilatory pathway improves the production of heterologous protein

**Fig. 6:**
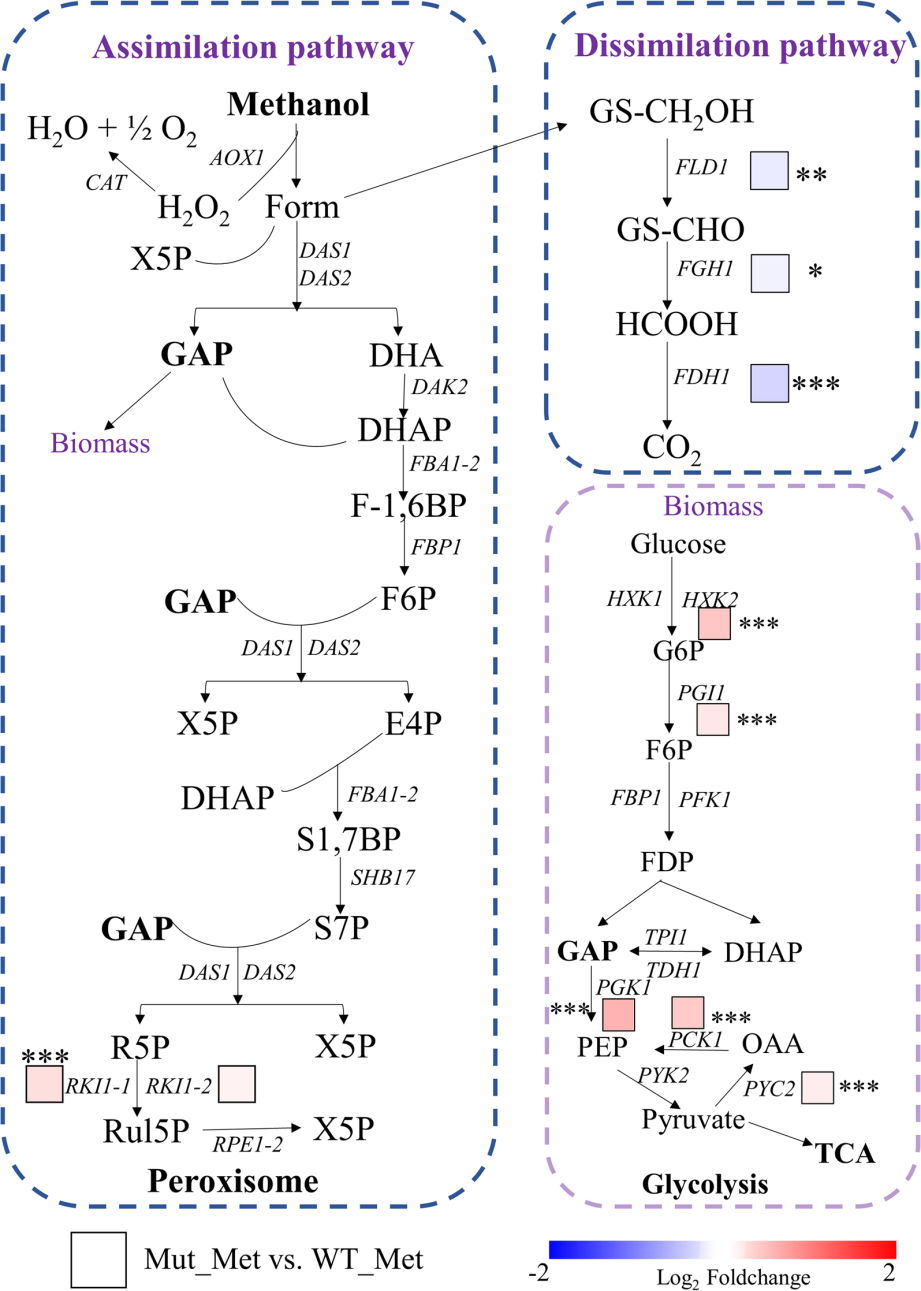
Expression levels of key genes. Comparative of the robust mutant with the wild type under identical methanol conditions. *AOX1*: alcohol oxidase 1; Form, formaldehyde; *DAS1*, dihydroxyacetone synthase; DHA: dihydroxyacetone; DHAP: dihydroxyacetone phosphate; E4P: erythrose-4-phosphate; *FBA1-2*: fructose 1,6-bisphosphate aldolase; *FDH1*: formate dehydrogenase 1; Form, formaldehyde; F1,6BP: fructose 1,6-bisphosphate; F6P: fructose-6-phosphate; GAP: glyceraldehyde-3-phosphate; R5P: ribose-5-phosphate; Rul5P: ribulose-5-phosphate; S1,7BP: sedoheptulose-1,7-bisphosphate; S7P: sedoheptulose-7-phosphate; X5P: xylulose-5-phosphate; GS-CH_2_OH: S-(hydroxymethyl)glutathione; GS-CHO: S-formylglutathione; HCOOH: formic acid; G6P: glucose-6-phosphate; FDP: fructose-1, 6-diphosphate; PEP: phosphoenolpyruvate; OAA: oxaloacetic acid; TCA: tricarboxylic acid cycle. *p < 0.05, **p < 0.01, ***p < 0.001

*Pichia pastoris* is a methylotrophic yeast, able to utilize methanol for energy production and to assimilate it as a sole carbon source for cell growth and producing target product [33]. The metabolism of methanol as part of carbon metabolism consists of two pathways, the assimilatory pathway and the dissimilatory pathway (Fig. 6). The assimilatory pathway of methanol is in peroxisomes of *P. pastoris* [24]. In the assimilatory pathway, methanol participates in the carbon metabolism to produce organic matters like endogenous and exogenous proteins. However, in the dissimilatory pathway, methanol is ultimately transformed into carbon dioxide. Mapping the results of RNA-seq into the methanol metabolism network, the dissimilatory pathway (*FLD1*, *FGH1*, and *FDH1*) of methanol was down-regulated in Mut_Met versus WT_Met, while some genes (*RKI1-1* and *RKI1-2*) involving in the assimilatory pathway were upregulated. The methanol in the robust mutant was more assimilated to generate biomass that genes (*HXK2*, *PGI1*, *PGK1*, *PCK1*, and *PYC2*) involved in glycolysis were significantly upregulated in comparison group Mut_Met versus WT_Met. These results indicated that the efficiency of the methanol assimilatory pathway may be improved to generate more product, which also explained the higher protein expression level in the robust mutant.

### Rational design to improve lipase expression level based on the overexpression of antioxidant genes

**Fig. 7:**
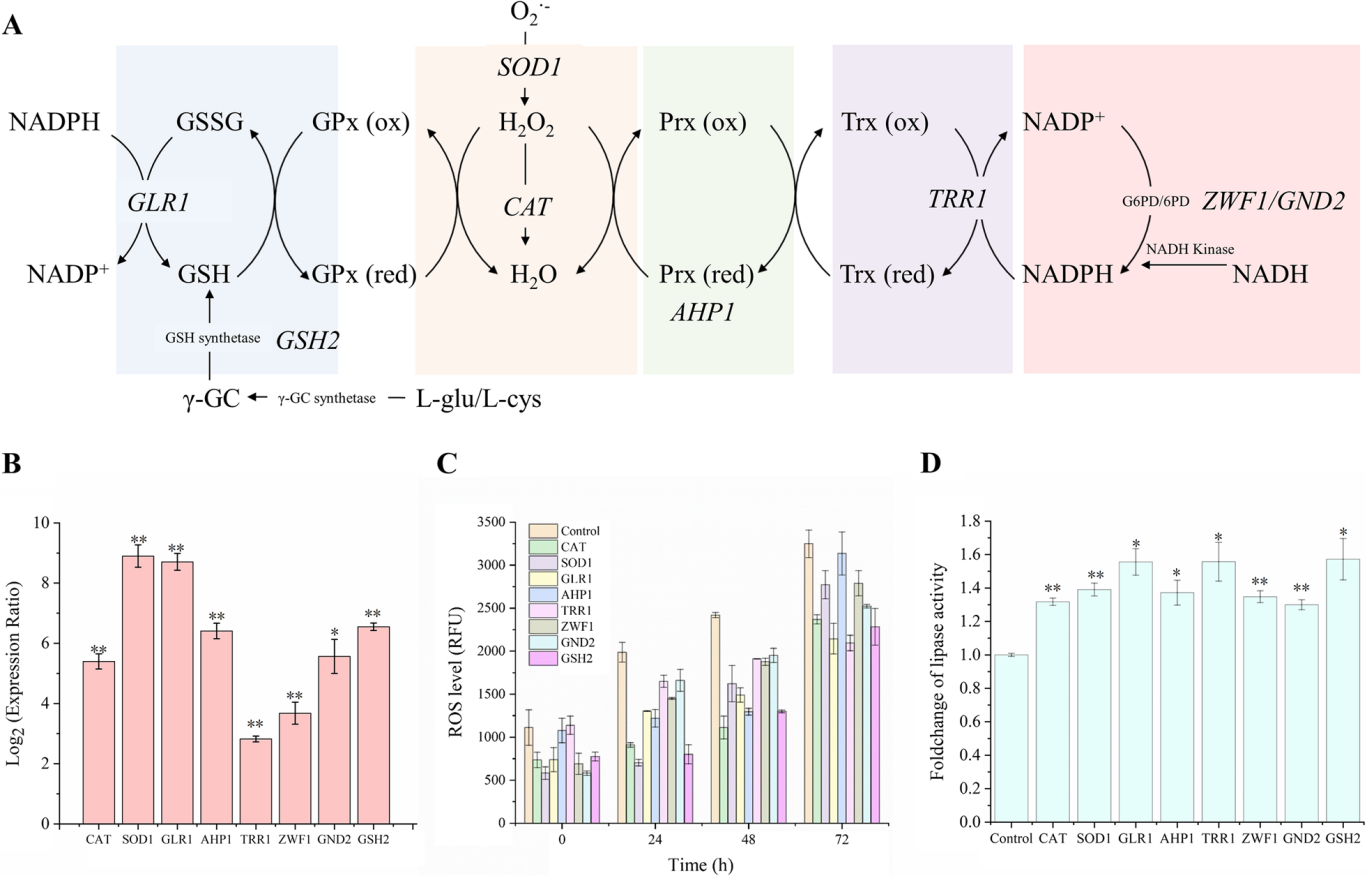
Overexpression of antioxidant genes to improve the heterologous protein expression ability of *P. pastoris*. **A** The antioxidant defence system of yeasts. O_2_^·−^: superoxide anion; SOD: superoxide dismutase; CAT: catalase; GPx: glutathione peroxidase; GLR1: glutathione reductase; Prx: peroxiredoxin/thioredoxin peroxidase; Trx: thioredoxin; TRR1: thioredoxin reductase; G6PD: glucose-6-phosphate dehydrogenase; 6PD: 6-phosphogluconate dehydrogenase; GSH: glutathione; γ-GC: gamma glutamylcysteine; l-glu: l-glutamic acid; l-cys: l-cysteine. **B** Relative transcription levels of antioxidant system-related genes. The expression ratio of a gene was analysed using the 2^−ΔΔCt^ method; **C** the intracellular ROS level of overexpressed strains and their blank control during methanol-induced fermentation; **D** foldchange of lipase activity levels in overexpressed strains. *p < 0.05, **p < 0.01

Based on the results above, we believed the engineering of the cell’s antioxidant defence system by overexpression of genes involved in endogenous antioxidants could alleviate the toxic effects induced by methanol, and thereby can increase the protein expression level in *P. pastoris*. According to the antioxidant defence system [34, 35] (Fig. 7A), *CAT*, *SOD1*, *GLR1*, *AHP1*, *TRR1*, *ZWF1*, *GND2,* and *GSH2* was solely overexpressed by constitutive promoter P_*GAP*_ in *P. pastoris* hosts. The *SOD1* gene encoding superoxide dismutase 1 converts superoxide anion (O_2_^·−^) to H_2_O_2_. The *CAT* encoding catalase directly degrades H_2_O_2_. Glutathione is one of the main antioxidants in living cells [36]. The *GLR1* encoding glutathione reductase helps to make reduced glutathione. The *GSH2* encoding glutathione (GSH) increases total glutathione content. In yeast cells, thioredoxins partner with peroxiredoxin in H_2_O_2_ signalling [34]. The *AHP1* encoding alkyl hydroperoxide reductase 1 is a member of the peroxiredoxin family to promote the elimination of cellular H_2_O_2_ [37]. The *TRR1* encoding thioredoxin reductase plays an important role in thioredoxin-dependent antioxidant pathways [38]. The reducing power provided by NADPH is generated from the pentose-phosphate pathway via glucose-6-phosphate dehydrogenase (encoded by *ZWF1*) and 6-phosphogluconate dehydrogenase (encoded by *GND2*) [39]. The relative transcription levels of these genes in gene-overexpressing strains were measured by RT-qPCR. As shown in Fig. 7B, these genes were successfully overexpressed.

To explore the contribution of antioxidant genes in lipase expression, flask-batch fermentation of each overexpressed strain was performed (Fig. 7C, D). Under the control of P_*AOX1*_, the lipase was expressed using methanol as an inducer. During the process of fermentation, the intracellular ROS levels of overexpression strains and the control were measured and compared. As shown in Fig. 7C, the ROS levels in these overexpressed strains were significantly lower than that of the blank control. It indicated that the contributions of antioxidant genes were very considerable. Compared to the blank control, the expression levels of lipase in overexpressed strains were improved up to 1.6-fold (Fig. 7D). These results revealed that overexpression of antioxidant genes improved the expression of lipase under methanol induction condition.

## Discussion

As we know, H_2_O_2_ is generated as a by-product of methanol metabolism, causing oxidative stress on *P. pastoris* cells [40]. The accumulation of ROS reduced the production of proteins. However, the response mechanism of *P. pastoris* to methanol is still unclear. Previously, we obtained a robust *P. pastoris* mutant with a strong ability to express heterogeneous proteins, which could be used as a good start point for revealing the underlying mechanism responding to oxidative stress under methanol induction. It is interesting to explore the differences in cellular response between the strains with different protein expression abilities. After RNA-seq, we found the GO terms related to redox were enriched in the comparison groups under methanol induction conditions versus glucose conditions (Fig. 3). Moreover, under methanol induction conditions, GO terms related to redox were also enriched in the robust mutant versus the WT. The results revealed that the robust mutant exhibited a lower level of ROS and enhanced protein production efficiency owing to its augmented oxidative stress tolerance. In addition, we improved the methanol-induced lipase expression ability of non-oxidative stress-tolerant strain by rationally increasing its ROS buffering capacity, further explaining the importance of strains with strong oxidative stress tolerance in methanol-induced fermentation.

Compared to the WT, we found that the antioxidant property of the robust mutant was mainly attributed to four characteristics: active redox processes, active environmental stress response (upregulation of MAPK pathway), higher expression levels of antioxidant-related transcription factors, and upregulation of ergosterol synthesis pathways. The results supported again the conclusion that intracellular redox balance is very important for the cell productivity of yeast [41]. The imbalance of redox will cause the accumulation of intracellular ROS, leading to protein peroxidation [42] and lipid peroxidation [43]. These disorders reduce the production of products in yeast. Hence, maintaining redox homeostasis in hosts will efficiently enhance their productivity. The antioxidant defence system in yeasts mainly consists of the enzymatic part and non-enzymatic part [35]. The key parts of the antioxidant defence system are shown in Fig. 7A. Superoxide dismutase, catalase, and peroxiredoxins are the main members of the enzymatic defence system. The non-enzymatic defence system generally includes GSH, thioredoxin, vitamin C (ascorbic acid), and other reducing agents. In addition, cofactor engineering for enhancing the production of products and improving cell growth has become popular. In cells, the pentose phosphate pathway is activated to compensate for the severe request of NADPH upon oxidative stress [44]. Besides, augmenting NADPH levels promoted protein production in *P. pastoris* [45].

As reported previously, it is an efficient way to enhance cell productivity by the overexpression of antioxidant genes. The production of ethanol was boosted by overexpressing multilevel defence system which effectively reduced the accumulation of cellular ROS and alleviated lipid peroxidation in *S. cerevisiae* [46]. A robust lipid production platform of *Yarrowia lipolytica* was built by modulation of oxidative stress defence pathway [47]. In this study, the overexpression of genes related to antioxidant defence system reduced the cellular ROS level during methanol-induced fermentation and then improved the production of lipase (Fig. 7). The researchers also found that the combination of effective genes can sometimes double the effect [46]. Therefore, in the follow-up of this study, we consider overexpressing the combination of these functional genes in *P. pastoris*, looking forward to obtaining strains with stronger protein expression potential.

The methods to improve the oxidative stress tolerance of yeast cells are not limited to simply overexpressing the genes encoding antioxidant enzymes. It is also a good way to start by repressing the source of ROS generation. Promisingly, this study revealed that the ergosterol pathway was significantly upregulated in strains under methanol condition versus glucose condition and the comparison group Mut_Met versus WT_Met (Fig. 4D). The increase in ergosterol content is conducive to inhibiting the production of intracellular ROS by NOX [48]. And, the supplementation of ergosterol mitigated oxidative stress in *S. cerevisiae* [49]. The mutants lacking ergosterol are hypersensitive to oxidative stress [50]. Moreover, Caspeta et al*.* [51] have proved that the ergosterol pathway is importantly related to the thermotolerance of yeast. Sterols including ergosterol are the main components of cell membranes. Adjusting the ratio of cell membrane components can also enhance cell tolerance [52]. Hence, the ergosterol synthesis pathway will be our future target to rationally enhance the oxidative stress tolerance of *P. pastoris*.

## Conclusions

This study found that a robust *P. pastoris* mutant with oxidative stress tolerance can express high levels of heterologous proteins under the induction of methanol. From the results of ROS levels and RNA-seq, we proved that methanol mainly induces the oxidative stress response in *P. pastoris*. Based on the importance of antioxidant defence system, we rationally improved the production of heterologous protein lipase up to 1.6-fold by overexpressing genes related to antioxidant defence system in *P. pastoris*. However, it is difficult to achieve the desired effect only by overexpression of a single functional gene. In the future, we will consider further improve the heterologous protein expression ability of *P. pastoris* from the view of global regulation. Our findings provide a further understanding of the impact of methanol on the expression of heterologous proteins in *P. pastoris* and highlight potential targets for constructing yeasts with high protein expression potential.

## Methods

### Strain and plasmid construction

The robust mutant was previously evolved by adaptive laboratory evolution [19]. Both the robust mutant and its WT expressed a recombinant protein lipase encoded by *RCL* gene and tightly regulated by the methanol-inducible promoter P_*AOX1*_. For gene overexpression, the antioxidant genes from the genome of *P. pastoris* were constructed into the pGAPZα vector using MultiF SeamLess Assembly Mix (ABclonal, China). The plasmids were introduced into *P. pastoris*-competent cells by electroporation, using an Eppendorf Eporator (Eppendorf, Germany).

### Expression of heterologous proteins

Under the control of inducible promoter P_*AOX1*_, the fermentation process was performed in BMGY/BMMY fermentation medium containing 100 mM potassium phosphate (pH 6.0), 1.34% yeast nitrogen base without amino acids, 4 × 10^−5^% biotin, and 1% glycerol (BMGY) or 0.5% methanol (BMMY) at 28 °C [16]. Methanol as a sole carbon source and inducer was supplied into the medium every 24 h. The fermentation broth of each sample expressing lipase was centrifuged, and aliquots of the supernatant were diluted to measure the lipase activities. The lipase enzyme activity of samples was assayed according to Sha et al*.* [16]. Under the control of constitutive promoter P_*GAP*_, the fermentation process of GFP was conducted in YPD liquid medium. The fermentation broth of each sample expressing GFP was centrifuged, and cell pellets were resuspended to measure fluorescence intensity at 485/528 excitement/emission (nm). The fluorescence intensity was normalized to 1 OD_600_.

### Measurement of ROS level

Intracellular ROS was measured using 2,7-dichlorodihydrofluorescein diacetate (DCFH-DA) [53] by three biological replicates of each sample. Briefly, the cells were harvested by centrifugation, resuspended in PBS, and treated in 10 μM DCFH-DA dissolved in DMSO for 1 h. After centrifugation, the cell pellets were collected and resuspended in PBS. After crushing by glass bead disruption, the supernatant was collected, and DCF fluorescence intensity was measured at 480/525 excitement/emission (nm). The protein concentration was measured by the Bradford method [54]. DCF fluorescence intensity was normalized to the protein level of the supernatant.

### RNA-sequencing

Samples for RNA-seq were collected from fermentation cultures at 60 h. Three biological replicates of each sample were adopted to ensure data reliability. The cDNA library construction and sequencing, quality control, and alignment to *P. pastoris* genome were provided by Novogene, Beijing, China (http://www.novogene.cn/). Genes with an adjusted *p*-value < 0.05 found by DESeq2 were assigned as differentially expressed. GO enrichment analysis was implemented by the Novogene cloud platform (https://magic.novogene.com) with adjusted *p*-value < 0.05 and log_2_ foldchange > 1.

### RNA isolation and RT-qPCR

The transcription levels of genes were analysed by RT-qPCR. Cells were collected and immediately frozen in liquid nitrogen. Total RNAs of samples were extracted by Yeast RNAiso Kit (Takara). For cDNAs synthesis, PrimeScript™ RT reagent Kit with gDNA Eraser (Perfect Real Time) from Takara was utilized with 1 μg of the total RNA as a template based on the manufacturer’s protocol. The reaction was terminated by heating at 85 °C for 5 s. The *actin* gene *ACT1* was selected as an endogenous control for the RT-qPCR to normalize the expression data for each gene. The primers for RT-qPCR used in this study are listed in Table 2. Amplification was performed using 2*SG Fast qPCR Master Mix (High Rox) (Sangon Biotech, China) in the ABI StepOne Plus Real-Time PCR System (ABI, Germany). The thermal cycling conditions for gene amplification were initial denaturation at 95 °C for 30 s, followed by 40 cycles each of denaturation at 95 °C for 3 s and annealing at 60 °C for 30 s. All experiments were independently performed in triplicate. The expression ratio of a gene was analysed using the 2^−ΔΔCt^ method [55]. Statistical analyses of data were performed using Microsoft’s Excel software.

**Table 2:** List of genes and primers for RT-qPCR used in this study

Name	Primer sequence (5ʹ-3ʹ)
*ACT1*	F: TATTGAAGTTGAAGCCCTCTGAGC R: CCTTCCGTGTGCAAATGAAACAC
*CAT*	F: TTCGACAACGCTAATCACGCTAAC R: TCACCTCAAACTCACCGAAAGCT
*SOD1*	F: TCGAACAATCCTCCGAAAGCAGC R: CCGTGGGTCTTACCAAATGGGT
*AHP1*	F: CTGGAGTCTTAGCTTGTGCTATTCC R: CGTCGATTTTCTCAATGAAAACAGG
*GLR1*	F: GGTGCAAAGACCCTTTTGATTGAAG R: GGTCCAGTTAAAGGAAAAATCGCC
*TRR1*	F: GGGTATGTTGGCTAACGGTATCG R: GGTCTCGGTAATGATCTCAGTGCC
*GSH2*	F: GCGATGTACCCCACCAATTTTGAG R: GCCCAGCCATTCCTGATTTTTGA
*ZWF1*	F: AGGGCGACGAGGACAAAGTTC R: GGGCAAAGCTAAGTAGAACAACCTG
*GND2*	F: GGTTTCACCGTCGTCGCTTACA R: CGGGATTACCAGCCTTGACCAATA
*Mxr1*	F: CCACAGTTGCAATACCAACAATCTC R: CGGAGAAGGAAGCTCTTCTTAGT
*Prm1*	F: ACAGGGACAATCTCTGAGTCTGA R: GTTGGAGCACTGTTGGGGTATAT
*Mit1*	F: GGTTCAAACTCTGGCAGTTCAGAT R: CCATTGGACATTCCATTGAAACCG

All ORF sequences can be found from http://PICHIAGENOME.org database

Forward (F) and reverse (R) primer sequences

## Data Availability

Not applicable.

## Abbreviations

WT: Wild type
ROS: Reactive oxygen species
ER: Endoplasmic reticulum
UPR: The unfolded protein response
PDI: Protein disulfide isomerase
GFP: Green fluorescence protein
AOX1: Alcohol oxidase 1
GAP: Glyceraldehyde 3-phosphate dehydrogenase gene
H_2_O_2_: Hydrogen peroxide
GEO: Gene Expression Omnibus
PCA: Principal component analysis
GO: Gene Ontology
BP: Biological process
CC: Cellular component
MF: Molecular function
NOX: NADPH oxidase
